# Effects of mistletoe products on pharmacokinetic drug turnover by inhibition and induction of cytochrome P450 activities

**DOI:** 10.1186/s12906-017-2028-1

**Published:** 2017-12-04

**Authors:** Michael Schink, Oliver Dehus

**Affiliations:** Helixor Heilmittel GmbH, 72348 Rosenfeld, Germany

**Keywords:** Cancer, Cytochrome P450, Helixor®, Interaction, Mistletoe, *Viscum album*

## Abstract

**Background:**

European mistletoe (*Viscum album*) products used in cancer therapy are frequently combined with other anti-cancer-drugs. Hence, potential herb-drug interactions have become a major safety concern in mistletoe therapy.

**Methods:**

Three European mistletoe products (Helixor® A, Helixor® M and Helixor® P from mistletoe grown on firs, apple trees and pines, respectively) were tested for inhibition of nine major cytochrome P450 (CYP) isoenzymes in a test system using pooled human liver microsomes and for induction of five CYP isoforms in human hepatocytes cultivated in vitro according to the relevant guideline.

**Results:**

Major inhibition did not occur in any of the CYP marker reactions. For some CYP isoenzymes, a minor or intermediate inhibition could be observed, but without dose effect relationship. Induction activity (≥ 1.5-fold increase) was not found with any of the three mistletoe products.

**Conclusion:**

Since no induction capacity was found and major inhibition above 50% did not occur even with the highest concentration used, which is approximately 100,000-fold higher than the clinically relevant dose in plasma, a clinically relevant herb-drug interaction is not expected for Helixor® A, M, and P.

## Background

European mistletoe products for injection made of *Viscum album* belong to the most frequently used herbal medicines in cancer patients in Central Europe, especially in Germany [1]. During anti-cancer treatment, they are usually combined with conventional cytostatic drugs and other supportive medicines. Such mistletoe products in particular proved to significantly increase the tolerability of chemotherapy by reducing therapy-related side effects and to improve the quality of life of cancer patients [2]. Phase I metabolism of many anti-cancer drugs used e.g. in chemotherapy or targeted therapy is mainly carried out via certain cytochrome P450 (CYP) isoenzymes [3]. A number of CYPs are subject to inhibition or induction by herbal compounds in vitro [4] indicating a potential risk for increased or decreased turnover of cytostatic drugs: for instance, the additional oral application of St. John’s wort (*Hypericum perforatum*) to chemotherapy with irinotecan not only deceased therapy-related myelosuppression, but also decreased the plasma levels of the active metabolite of the anti-cancer drug [5]. Thus, the potential interference with the metabolism of cytostatic drugs has become a major safety concern also in mistletoe therapy and needs to be elucidated. For this purpose, we investigated the in vitro potential of the mistletoe products Helixor® A, M and P to induce or inhibit CYP isoenzymes in accordance with the relevant guideline [6]. The results are discussed in the context with the pertinent scientific literature on interactions between mistletoe products and cytostatic drugs.

## Methods

### Mistletoe products

Three solutions for injection, produced by Helixor Heilmittel GmbH, Rosenfeld, Germany, were tested (Helixor® A, batch no. 040263, Helixor® M, batch no. 030954 and Helixor® P, batch no. 030902). All products are manufactured from 5% (*w*/*v*) aqueous summer and winter extracts from fresh mistletoe herb (*Viscum album* L.). The three different sorts (Helixor® A, M and P) represent three different host trees: Helixor® A from fir (*Viscum album* L. subspecies *abietis*), Helixor® M from apple tree (*Viscum album* L. subspecies *album*) and Helixor® P from pine (*Viscum album* L. subspecies *austriacum*). There are clear differences between these three Helixor® sorts in the content and spectrum of various active constituents, e.g. mistletoe lectins, and also in cytotoxic activity [7]. From all three Helixor® sorts, the highest available strength (50 mg each) of the respective product was chosen as test item.

### Human liver microsomes

Human liver microsomes (20 mg/ml) were purchased from In Vitro Technologies, Inc. Baltimore, USA as pooled, mixed gender human liver microsomes, art. no. X00821, lot no. MGU, delivered on dry ice and stored at −80 °C until use.

### Human hepatocytes

Freshly isolated, pre-plated human primary hepatocytes from five different donors (3 female, 2 males, lot nos. 041202 HU 063, 041208 HH 069, 041213 HH 070 HP, 050203 HRP 005, and 050210 HLP 006) were supplied by Cytonet GmbH & Co. KG, Weinheim, Germany and used for the enzyme induction assays.

### Marker substrates

7-Ethoxyresorufin, content 99.0 / 100%, lot. No. 021 K4024 (inhibition test) and 033 K4058 (induction test), Sigma; coumarin, content 99.9%, lot no. 90 K3684, Sigma; S-mephenytoin, content 99.0% lot no. P6178, Biomol; paclitaxel, content 99.3%, lot. No. 3508F, ICN; diclofenac sodium, content >99%, lot. No. 107H4748, Sigma; bufuralol hydrochloride salt, content 99.5%, lot. No. 102–155, Ultrafine; chlorzoxazone, content 99.6%, lot. No. S08663–121, Aldrich; testosterone, content 99.9%, lot. No. S542, Applichem.

### Reference inhibitors

Furafylline, purity 98.9%, lot. No. 063 K4702, Sigma; 8-methoxypsoralene, purity >98%, lot no. 30122, ICN; triethylenethiophosphoramide, purity 100.9%, lot no. 081 K1195, Sigma; ketoconazole purity 99.3%, lot. No. 78353, ICN; sulfaphenazole purity 99.1%, lot. No. 3G03777, Applichem; omeprazole, purity 100%, lot. No. 021 K1605, Sigma; quinidine sulphate, purity 99.3%, lot. No. 4353B, ICN; diethyldithiocarbamic acid sodium salt, purity 100%, lot. No. 110 K2615, Sigma.

### Reference inducers

Omeprazole, purity 100%, lot no. 021 K1605, Sigma; phenobarbital sodium, purity 99.0%, lot no. 076H0292, Sigma, ethanol, purity 99.9%, lot no. 3006761, AppliChem, rifampicin, purity 99.6%, lot no. 304324/1, Fluka.

### Cytochrome P450 inhibition

The mistletoe products were tested for effects on marker reactions specific for CYP 1A2, 2A6, 2B6, 2C8, 2C9, 2C19, 2D6, 2E1, and 3A4. Final concentrations of 0.5, 0.005, and 0.0005 mg/ml were used. Incubation was carried out in 96-well microtiter plates at 37 ± 0.5 °C in the presence of 2 mM NADPH and 3.3 mM magnesium chloride with pooled human liver microsomes (0.5 mg microsomal protein/ml) in 50 mM potassium phosphate buffer, pH 7.4. All tests were performed in triplicate. The total volume of the individual incubation was 200 μl. The marker substrate concentrations in the incubation mixtures were chosen at concentrations close to their apparent K_m_. The test items and reference inhibitors were preincubated with microsomal suspension for 5–10 min (CYP 1A2, 2C19) or 11.5–12.5 min (all others CYP isoenzymes), respectively, and the incubations were started by addition of the respective marker substrate. The incubation was stopped after 30 ± 1 min by addition of stop reagent (150 μl 100% acetonitrile or 50 μl 43% phosphoric acid for CYP2E1), centrifuged for 10 min at 4 °C and approximately 3000 x g, and the supernatant was transferred to a new 96-well microtiter plate for marker reaction specific analysis of remaining marker substrates and resulting metabolites.

Marker reactions containing no test item and no CYP specific inhibitor but identical concentrations of the corresponding solvent type of the test item incubations served as negative controls of inhibition (NC). Marker reactions containing a marker reaction specific reference inhibitor instead of one of test items served as positive controls of inhibition (PC).

The final concentrations of the marker substrates in the respective incubation media were as follows: 5 μM 7-ethoxyresorufin for CYP1A2; 5 μM coumarin for CYP2A6, 200 μM S-mephenytoin for CYP2B6; 10 μM paclitaxel for CYP2C8; 10 μM diclofenac for CYP2C9; 50 μM S-mephenytoin for CYP2C19; 20 μM bufuralol for CYP2D6, 50 μM chlorzoxazone for CYP2E1; 100 μM testosterone for CYP3A4.

The measured substrate specific metabolites were resorufin for CYP1A2; umbelliferone for CYP2A6; nirvanol for CYP2B6; 6α-hydroxy-paclitaxel for CYP2C8; 4′-hydroxy-diclofenac for CYP2C9; 4′-hydroxy-mephenytoin for CYP2C19; hydroxybufuralol for CYP2D6; 6′-hydroxy-chlorzoxazone for CYP2E1; 6β-hydroxy-testosterone for CYP3A4.

The following final concentrations of CYP specific inhibitors in the incubation samples were used: 25 μM furafylline for CYP1A2; 0.75 μM 8-methoxypsoralen for CYP2A6; 75 μM triethylenethiophosparamide for CYP2B6; 25 μM ketoconazole for CYP2C8; 2 μM sulfaphenazole for CYP2C9; 7.5 μM omeprazole for CYP2C19; 0.3 μM quinidine for CYP2D6; 30 μM diethyldithiocarbamic acid sodium salt for CYP2E1; 0.15 μM ketoconazole for CYP3A4.

### Cytochrome P450 induction

For evaluation of CYP induction activities of the mistletoe products, freshly isolated human primary hepatocytes from three different donors were used. 2–5 × 10^5^ cells were plated on collagen-coated 24-well plates in medium optimized for hepatocytes (HIM) containing 100 IU/ml penicillin, 100 μg/ml streptomycin and 0.05 μMol dexamethasone. After addition of test item or reference inducer, plates were cultivated for 48 ± 4 h under the following incubation conditions: 37 ± 2 °C, 5% CO_2_, approximately 95% humidity. Three incubation mixtures were prepared per concentration per test item, positive and negative control per CYP isoenzyme. Medium (containing test reagent where applicable) was changed once after 24 ± 2 h. All wells were checked by microscopic analysis for contamination and cell morphology before incubation with test item, positive / negative controls and at the end of incubation. After the induction phase the supernatants were removed and replaced by medium supplemented with 3 mM salicylamide (S-HIM). After 10 min incubation at 37 °C the supernatants were replaced by 350 μl/well S-HIM containing the respective concentration of the specific substrate for the enzyme marker reactions. After further 3 h ± 15 min of incubation the supernatants of all samples were collected in 2 ml tubes which contained 350 μl ice-cold 100% acetonitrile or 70 μl of 43% phosphoric acid for the CYP2E1 incubation. Precipitated protein was sedimented by centrifugation for 10 min at 4 °C and approximately 14,000 rpm. 200 μl of the supernatants were transferred to microtiter plates for marker reaction specific quantification of remaining marker substrates and resulting metabolites.

Helixor® A, M and P were added at final concentrations of 10 μg/ml, 5 μg/ml and 0.5 μg/ml each. Hepatocyte incubations containing no test item but the respective reference inducers served as positive controls (PC). The following final concentrations were used: 50 μM omeprazole for CYP1A2; 1 mM phenobarbital sodium for CYP2B6 and CYP2C9; 100 mM ethanol for CYP2E1; 10 μM rifampicin for CYP3A4. Incubations without test item or reference inducers in medium containing 0.5% water served as negative controls (NC) for incubations with Helixor® A, M and P.

The final concentrations of the marker substrates in the respective incubation media were as follows: 2 μM 7-ethoxyresorufin for CYP1A2; 1 mM S-mephenytoin for CYP2B6 (100/200 μM for the first three donors. Since metabolite concentration was below level of quantification the higher concentration was used for the following donors); 50 μM diclofenac sodium for CYP 2C9; 200 μM chrorzoxazone for CYP 2E1; 200 μM testosterone for CYP 3A4.

### Fluorescence based sample analysis

The marker metabolite of CYP 1A2 (resorufin) was quantified by fluorescence based sample analysis (ex: 544 nm, em.: 590 nm) using a Fluoroskan Ascent microplate fluorometer (Labsystems, Finland).

### Tandem HPLC-mass spectroscopy

For the assessment of effects on the CYP isoenzymes 2B6, 2C9, 2C19, 2E1, and 3A4 (induction or inhibition) as well as 2A6, 2C8 and 2D6 (inhibition only) the respective marker substrates and marker metabolites were separated and detected by use of HPLC (Waters Alliance HT 2790, Eschborn, Germany) and mass spectroscopy (Quattro Micro, Micromass, Wythenshawe, UK) on a C18 column (Luna 3u C18(2), 30 × 2.0 mm, 3 μm, Phenomenex Aschaffenburg, Germany). For CYP 2E1 a C8 column (Luna C8 (2), 150 × 2.0 μm, 5 μm) was used. Further HPLC parameters are summarized in Table 1.

**Table 1:** HPLC / MS parameters for marker substrate and marker metabolite separation and detection

CYP Iso-enzyme	Marker substrate	Marker metabolite	Gradient/isocratic	Injection volume [μl]	Flow rate [ml/min]	Column temp. [°C]	Stop time [min]	Detection
2A6	coumarin	umbelliferone	0–6 min linear	10	0.4	30	8	Transition (m/z) 163.0 > 107.0
2B6	S-mephenytoin	nirvanol	0–4 min linear	10	0.4	30	6	Transition (m/z) 205.0 > 134.0
2C8	paclitaxel	6α-hydroxypaclitaxel	0–6 min linear	10	0.4	30	7.5	Transition (m/z) 870.2 > 524.9
2C9	diclofenac	4′-hydroxydiclofenac	0–6 min linear	10	0.4	30	7	SIR (m/z) 265.9 / 249.9
2C19	S-mephenytoin	4′-hydroxymephenytoin	0–6 min linear	10	0.4	30	6	Transition (m/z) 235.0 > 150.2
2D6	bufuralol	hydroxybufuralol	0–6 min linear	10	0.4	30	8	MRM (m/z) 278.2 > 186.1
2E1	chlorzoxazone	6-hydroxychlorzoxazone	Isocratic	20	0.5	30	8	Absorption at 298 nm
3A4	testosterone	6β-hydroxytestosterone	0–6 min linear	10	0.4	30	7.5	Transition (m/z) 305.0 > 287.0

*m/z* mass-to-charge ratio, *SIR* selected ion recording, *MRM* multiple reaction monitoring

### Data analysis and acceptance criteria of CYP induction and inhibition tests

Data analysis of the CYP induction experiments was performed using standard software: MS-EXCEL™ (Microsoft Corp., Redmond, USA), MassLynx™ V3.5and QuanLynx™ (Micromass Ltd., Wythenshawe, UK), Ascent Software V2.4.2 (Thermo Labsystems, Milford, USA), and ChemStation Rev.A.09.01 (Agilent Technologies, Santa Clara, USA). The mean of n replicates, standard error of mean, % compared to negative control, and standard error were calculated using the respective Excel-functions.

In those cases where fluorescence was detected using a micrometer plate reader, the blank was substracted from the means before further calculations. Based on the means the percentage peak areas or fluorescence units compared to the negative control were derived. Additionally to standard blank samples, a test item interference control sample (blank sample containing the highest test item concentration but without the respective marker substrates) was used for fluorescence analysis in order to detect effects exhibited by the test item possibly interfering with the fluorescence based sample analysis.

In the incubation experiments with human microsomes the mean and the standard error of mean of the signals (e.g. peak areas, relative fluorescence units) of the replicate incubation per test item concentration or per control at the end of the incubation time were determined as measures of the relative activity of the respective test system.

The relative standard error of mean of the qualifier peak areas should be less than 20%. In those cases, where sample sequences contained less than four qualifiers, the difference between the lowest and highest qualifier values were evaluated and accepted if the difference was less than 30%. Otherwise the analysis of this series was repeated. In the case of the CYP inhibition experiments the data was accepted only if the positive control of inhibition inhibited the respective marker reaction at least 30% compared to the respective negative control of inhibition.

The definition of major, intermediate and minor inhibition was an increase of marker reaction activity of >50%, 25–50% and <25%, respectively; the definition for induction was a ≥ 1.5-fold increase in marker reaction activity compared to the negative control.

## Results

### Inhibition of CYP marker reactions in human liver microsomes

**Fig. 1:**
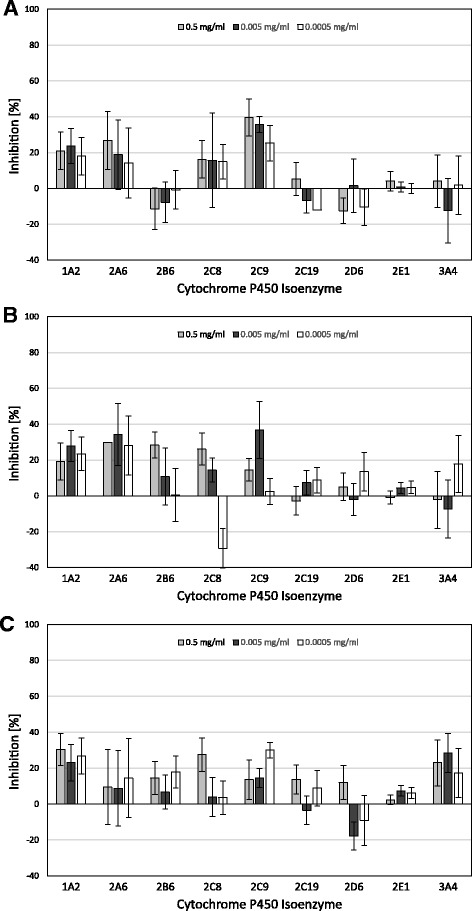
Inhibition of CYP Marker Reactions in Human Liver Microsomes by Helixor® A (**a**), Helixor® M (**b**), and Helixor® P (**c**) Effects of the mistletoe products on the metabolic activity of nine major human hepatic cytochrome P450 isoenzymes at 0.5 mg/ml (first bar), 0.005 mg/ml (second bar), and 0.0005 mg/ml (third bar)

Under the conditions used in this study, none of the test items exhibited major inhibition (above 50%) of any of the CYP marker reactions. Intermediate inhibition (from 25 to 50%) was observed for Helixor® A with CYP2A6 and CYP2C9 (Fig. 1a), for Helixor® M with CYP1A2, CYP2C8, CYP2A6, CYP2B6 and CYP2C9 (Fig. 1b) and for Helixor® P with CYP1A2, CYP2C8, CYP2C9 and CYP3A4 (Fig. 1c). No dose effect relationship could be observed. In all other tests no or minor inhibition (less than 25%) occurred.

### Induction of CYP isoenzymes in human hepatocytes

Hepatocytes incubated with Helixor® A demonstrated no changes in cell morphology compared to negative control. Incubation with Helixor® M at 10 μg/ml and with Helixor® P at 5 μg/ml and 10 μg/ml led to minor changes in cell morphology of hepatocytes from some donors. Change in morphology was accompanied with a partial detachment of cells from substrate and is likely due to a moderate cytotoxic effect exerted by Helixor® A and P at the highest concentrations.

The criterion for an induction of the tested CYP isoenzymes (an at least 1.5-fold increase in marker reaction activity compared to the negative control) was not met by any of the test items at any of the concentrations for all cytochromes P450 isoenzymes tested (Table 2). All test mixtures responded to the respective control inducers as positive controls (PC) with the exception of ethanol as the reference inducer of CYP2E1. It is well known and described in literature that CYP2E1 is hardly inducible and a reliable inducer of CYP2E1 is not described in literature so far. However, it is assumed that a strong inducing substance would lead to an elevated level of marker substrate activity in the present test system.

**Table 2:** Induction of different cytochrome P450 isoenzymes in cultured human hepatocytes from five different donors with Helixor® A (HA), Helixor® M (HM) and Helixor® P (HP). Fold induction of activity over respective negative control (incubation with no test item) is given. Test mixtures containing reference inducers were used as positive controls (PC)

CYP	CYP1A2			CYP2B6			CYP2C9			CYP2E1			CYP3A4		
Donor	D1	D2	D3	D1	D4	D5	D1	D4	D5	D1	D2	D3	D1	D2	D3
PC	19.2	21.8	31.6	3.0	3.7	2.9	1.5	1.3	1.5	1.2	1.0	1.0	1.7	1.9	3.3
HA 10 μg/ml	0.5	0.8	0.4	1.0	0.6	1.2	0.6	0.6	0.4	0.9	0.6	0.8	1.0	0.7	0.9
HA 5 μg/ml	0.6	1.1	0.4	1.0	0.9	1.2	0.6	1.1	1.0	1.0	0.6	1.0	1.4	0.8	0.9
HA 0.5 μg/ml	0.8	1.2	0.4	1.0	1.1	0.8	0.9	1.2	0.4	0.9	0.7	1.0	1.4	0.9	1.1
HM 10 μg/ml	0.6	0.8	0.3	0.6	0.2	0.9	0.5	0.1	0.8	0.9	0.7	0.8	0.7	0.4	0.4
HM 5 μg/ml	0.6	1.0	0.4	1.2	0.8	1.2	0.8	0.7	0.9	1.1	0.9	1.3	1.1	0.5	0.9
HM 0.5 μg/ml	0.8	1.1	0.4	1.3	1.2	1.0	0.9	1.0	1.0	1.1	0.8	1.3	1.4	0.6	1.1
HP 10 μg/ml	0.5	0.3	0.1	0.4	n.d.	0.4	0.4	n.d.	0.1	0.3	0.1	n.d.	0.4	0.1	0.1
HP 5 μg/ml	0.7	0.6	0.4	0.5	0.0	0.9	0.7	0.0	0.2	0.8	0.4	0.6	0.5	0.2	0.3
HP 0.5 μg/ml	0.8	0.9	0.3	1.3	0.7	1.1	1.1	1.3	1.3	0.9	0.9	1.2	1.0	0.6	1.0

D1, D2, D3, D4, D5: donor 1 to donor 5;

Positive controls (PC): 50 μM omeprazole (1A2); 1 mM phenobarbital (2B6, 2C9); 100 mM ethanol (2E1); 10 μM rifampicine (3A4);

*n.d.* not detectable

## Discussion

The aim of the studies was to investigate the in vitro potential for induction or inhibition by the three commercially available mistletoe products Helixor® A, M and P of clinically relevant cytochrome P450 isoenzymes.

Induction of CYP marker reactions was tested in freshly isolated human hepatocytes with respect to the CYP isoenzymes 1A2, 2B6, 2C9, 2E1, and 3A4. At the highest concentration (10 μg/ml) Helixor® P and partly Helixor® M showed a moderate cytotoxic effect on hepatocytes of some donors. In this respect, 10 μg/ml was the highest concentration employable in the induction studies with the test items, which is approximately 2000-fold higher than the clinically relevant dose in plasma.

The results of the marker reaction experiments indicated no induction potential of all the Helixor® products on the five CYPs investigated at any of the concentrations tested. Marker reaction activity in hepatocytes incubated with the test items was in the range of the negative control for all donors and all CYPs tested.

The experiments on inhibitory effects of Helixor® A, M and P on the enzymatic activity of nine CYP isoforms in human liver microsomes yielded no major inhibition (above 50%) with any of the CYP marker reactions, even with the highest concentration of 500 μg/ml. Furthermore, for the CYP isoenzymes either minor or intermediate inhibition could be observed but with no dose effect relationship. Putative increase as well as decrease of inhibition of the corresponding marker reactions with increasing concentrations of test item was observed for various CYPs. Thus, the mentioned putative dose dependent effects are most likely due to statistical variations of approximately 20% and cannot be regarded as inhibition. It has also to be considered that inhibition rates of approximately 20% are likely irrelevant in a biological (in vivo) system.

These findings regarding inhibitory and stimulatory effects of the Helixor® products confirm and extend the results of another in vitro study on the capacity of Helixor® M and three other mistletoe products to induce the metabolic activity in the liver cell line HepG2 [8]. For evaluation of phase I reactions the 7-ethoxy-resorufin-O-deethylation assay (specific for cytochrome P450 1A1 and 1A2) and the N-demethylation of aminophenazone (specific for CYP 3A1 and 3A2) were used. Phenobarbital and dexamethasone were included as reference inducers, respectively. None of the mistletoe products was able to induce an increase of either the CYP 1A1 and 1A2-specific or the CYP 3A1 and 3A2-specific marker reactions.

The results presented here are also in agreement with the results of very similar investigations on three other mistletoe products [9]. These products, albeit produced in a clearly different way than the Helixor® products [10], also showed no or minor potential for herb-drug interactions by interference with CYP isoenzymes. In a further study Engdal and Nilsen reported a weak inhibition of cytochrome P450 3A4 with another, fermented mistletoe product but at very high dosages, far beyond the anticipated systemic concentrations under therapeutic conditions [11]. 100 μg/ml of the mistletoe product, being the highest applicable dose in the experiment due to limitations in solubility, exhibited only moderate inhibition below 50%. Therefore, clinically relevant systemic interactions with the CYP isoenzymes were considered unlikely. Nevertheless, mistletoe is repeatedly mentioned in overviews on potential interactions of herbal products with anti-cancer drugs [4, 12], possibly due to an overvaluation of the results of Engdahl and Nielsen.

In fact, demonstration of significant effects of herbal compounds on CYP isoenzymes in vitro usually causes concerns about interactions of those drugs with cytostatic therapies. However, such concerns often cannot be confirmed in vivo. Milk thistle (*Silybum marianum*) and grapefruit juice for example both proved to inhibit CYP3A4 isoenzyme in vitro [13, 14]. But only the latter was shown to induce an increase of the systemic exposure of important cytostatic products (e.g. erlotinib, everolimus, lapatinib, sunitinib and other drugs) if consumed simultaneously [15]. In contrast additional application of milk thistle exhibited no measurable effect on pharmacokinetics with irinotecan, which is mainly metabolized via CYP3A4 [16] as well as on CYP3A4/5 and further CYP isoenzymes as shown in healthy volunteers [17]. On the other hand St. John’s wort (*Hypericum perforatum*) decreased the plasma levels of the active metabolite of irinotecan indicating stimulation rather than inhibition of CYP3A4 and other major metabolism pathways [5] whereas in vitro investigations also demonstrated inhibitory activities of constituents of *Hypericum* on CYP3A4 and other CYP isoenzymes [18]. Therefore, even if stimulatory or inhibitory effects could be shown in vitro a herbal drug may not exhibit a comparable effect during clinical application.

As shown in the present study, Helixor® mistletoe products seem to have no potential for induction or inhibition of the cytochrome P450 enzyme system even in vitro. Since CYP isoenzymes in particular play a critical role in the metabolism of a large number of anti-cancer drugs currently used for chemotherapy (e.g. taxanes, cyclophosphamide, doxorubicin and vinblastine) [4] it is likely that no clinically important interactions are to be expected for Helixor® A, M and P in vivo under therapeutic conditions.

This conclusion is supported by the results of several clinical studies. A phase I trial investigated the impact of an additional therapy with Helixor® A to gemcitabine in patients with far advanced tumors [19]. In this study Helixor® A was subcutaneously administered daily up to a dose of 250 mg. The addition of the mistletoe product did not affect the pharmacokinetics of gemcitabine at any of the mistletoe dose levels tested, suggesting that mistletoe can be added to gemcitabine without concern about adversely affecting metabolism of the cytostatic drug.

In a randomized controlled trial the effects of an additional therapy with the mistletoe products Helixor® or Iscador® during chemotherapy were assessed at the end of the treatment showing significantly better tolerance of chemotherapy and an increase in quality of life in the verum groups as compared to the untreated controls [20, 21]. The anti-tumoral effects of the treatment regimens (chemotherapy plus Helixor® or Iscador® vs. chemotherapy alone) were then further monitored during a five years follow up period indicating no influence of an additional mistletoe treatment on the frequency of relapse or metastasis during the observation period and thus on the efficiency of chemotherapy [22, 23].

Finally, in a non interventional study gynecological outdoor patients were interviewed with a standardized questionnaire on use of CAM. The authors concluded that interactions of mistletoe products with conventional drugs are unlikely when used in addition to chemotherapy or endocrine therapy. Only in case of antibodies the risk for interactions with mistletoe therapy was classified possible due to theoretical considerations while it was stated that no data regarding direct interactions or interactions via metabolism by the cytochrome P450 systems are available [24]. Meanwhile, first experiences with a simultaneous application (same-day intravenous infusions) of Helixor® mistletoe products and monoclonal antibodies (mAb) including bevacizumab, cetuximab, and trastuzumab have been published, suggesting that the combined treatment with mistletoe products and mAb is safe [25].

These results corroborate the assumption that better tolerance of chemotherapy and putatively also of other oncological treatments due to additional mistletoe treatment may not be accompanied by a decreased anti-tumor efficiency of the conventional therapies as there is no evidence for pharmacologically and clinically relevant interactions.

## Conclusions

For the clinically most relevant CYP isoenzymes 1A2, 2A6, 2B6, 2C8, 2C9, 2C19, 2D6, 2E1 and 3A4, no induction capacity (i.e. an at least 1.5-fold increase of marker reaction activity) was found. Furthermore, no major inhibition above 50% of marker reaction activity occurred even with the highest concentration of the mistletoe products, which is approximately 100,000-fold higher than the therapeutic dose in plasma. Thus, a clinically relevant herb-drug interaction involving CYP isoenzymes is not to be expected for Helixor® A, M, and P.

## Abbreviations

CAM: complementary and alternative medicine
CYP: cytochrome P450
FDA: US food and drug administration
HPLC: high performance liquid chromatography
m/z: mass-to-charge ratio
MRM: multiple reaction monitoring
MS: mass spectrometry
NC: negative control
PC: positive control
SIR: selected ion recording
